# Cornea nerve fibre state determines analgesic response to tapentadol in fibromyalgia patients without effective endogenous pain modulation

**DOI:** 10.1002/ejp.1435

**Published:** 2019-06-24

**Authors:** Tine van de Donk, Monique van Velzen, Albert Dahan, Marieke Niesters

**Affiliations:** ^1^ Department of Anesthesiology Leiden University Medical Center Leiden the Netherlands

## Abstract

**Background:**

Tapentadol is a centrally acting analgesic with μ‐agonistic activity combined with noradrenaline reuptake inhibition. Its mechanism of action relies on improvement of descending pain inhibition. In the current study, tapentadol's ability to enhance conditioned pain modulation (CPM, an experimental measure of descending pain inhibition) was evaluated in fibromyalgia patients with absent or reduced CPM responses.

**Methods:**

A total of 34 fibromyalgia patients completed this double‐blind trial. Patients were randomized to receive treatment with tapentadol sustained‐release or placebo for a 3‐month period with 1‐month follow‐up. At baseline, the cornea nerve fibre state (CNFS) was quantified to determine the presence of nerve fibre pathology and assess its value in the prediction of the analgesic response.

**Results:**

Tapentadol significantly increased CPM responses during treatment with an average increase from baseline of 20.5 ± 12.5% (tapentadol) versus 3.0 ± 11.2% (placebo; *p* = 0.042). No treatment effect was observed for the absolute pain scores, however, analgesia responder rate analyses demonstrated a treatment effect in favour of tapentadol. Pain relief (a reduction in pain score ≥ 30%) was predicted by the presence of a normal CNFS (*p* = 0.035). Patients with an abnormal CNFS had no analgesic effect from tapentadol despite an increase in CPM.

**Conclusions:**

In chronic pain patients with fibromyalgia, the increase in endogenous pain inhibition by tapentadol was translated into analgesia in patients with a normal CNFS. In those with abnormal CNFS, tapentadol treatment was without analgesic effect.

**Significance:**

In this double‐blind randomized placebo‐controlled trial, we showed that tapentadol significantly enhanced the descending pain inhibition in fibromyalgia patients. Tapentadol‐induced pain relief was only present in patients with a normal CNFS.

## 1 INTRODUCTION

Tapentadol is a combined μ‐opioid receptor agonist and noradrenaline reuptake inhibitor (Frampton, 2010; Steigerwald, Müller, Kujawa, Balblanc, & Calvo‐Alén, 2012; Zajączkowska et al., 2018). The involvement of both opioidergic and adrenergic pathways is associated with modulation of the endogenous pain system (Schröder, Vry, Tzschentke, Jahnel, & Cristoph, 2010; Zajączkowska et al., 2018). This modulatory system is an important regulator of normal perception of pain and engages either facilitatory or inhibitory pathways that interact (enhance or suppress) with afferent nociceptive input at the level of the spinal cord dorsal horn (Ossipov, Morimura, & Porreca, 2014). An imbalance between these opposing modulatory systems has been related to several chronic pain syndromes (Lewis, Rice, & McNair, 2012). We previously showed that tapentadol treatment in patients with chronic pain from diabetes‐induced polyneuropathy enhanced descending inhibition, as measured by the experimental paradigm of conditioned pain modulation (CPM), an effect that was correlated to tapentadol's analgesic efficacy (Niesters et al., 2014).

In the current study, we evaluated the ability of tapentadol to enhance descending inhibition in chronic pain patients with fibromyalgia with reduced CPM responses at baseline, and further assessed whether the improvement of descending inhibition was associated with reduced pain reporting. Fibromyalgia is a chronic pain syndrome characterized by widespread pain, often accompanied by a range of secondary symptoms including fatigue, depression and several cognitive and somatic disturbances (Clauw, 2014). So far, there is no clear pathophysiological substrate to explain the fibromyalgia syndrome. The most accepted hypothesis is that fibromyalgia originates at central sites. Evidence for this comes from observations of increased neuronal activity during non‐noxious stimulation in brain regions involved in pain perception and decreased activity of the descending inhibitory pain pathway (Clauw, 2014; O'Brien, Deitos, Triñanes Pego, Fregni, & Carillo‐de‐la‐Peña, 2018; Schmidt‐Wilcke & Clauw, 2011).

Recent evidence suggests that the peripheral nervous system may additionally be involved in the pathophysiology of fibromyalgia. Abnormalities in peripheral C‐fibres are deduced from the results of quantitative sensory testing and observed in skin biopsies and in the upper layer of the cornea. The cornea contains a large number of C‐fibres that can be visualized with cornea confocal microscopy (CCM) (Caro & Winter, 2014; Doppler, Rittner, Deckart, & Sommer, 2015; Giannoccaro, Donadio, Incensi, Avoni, & Liguori, 2014; Oaklander, Herzog, Downs, & Klein, 2013; Oudejans et al., 2016; Ramirez et al., 2015; Turan et al., 2018). The CCM technique allows for sensitive and reproducible evaluation of the presence of peripheral neuropathy and is highly correlated to nerve counts in skin biopsies (Jiang, Yuan, Gu, & Zhuang, 2016; Petropoulos et al., 2013; Tavakoli et al., 2010). We and others recently showed that approximately 50% of fibromyalgia patients have small fibre disease as objectified from reduced density of C‐fibres in the skin or cornea (Oudejans et al., 2016; Ramirez et al., 2015; Turan et al., 2018).

In the current study, we hypothesize that tapentadol improves descending inhibition and induces pain relief. We aim that tapentadol reactivates CPM responses and that these responses are associated with reduced pain reporting. Furthermore, CCM testing was performed at baseline to assess the prevalence of small fibre disease in our fibromyalgia population and to assess whether a possible improvement in pain reporting may be related to the cornea nerve fibre state (CNFS).

## 2 METHODS

### 2.1 Ethics and protocol registration

This single center, double‐blind, randomized placebo‐controlled trial was performed at the Anesthesia & Pain Research Unit of the Department of Anesthesiology of the Leiden University Medical Center. The study protocol was approved by the local Committee on Medical Ethics and the Central Committee on Research Involving Human Subjects in The Hague. From all patients included in the study, oral and written informed consent was obtained after written information was provided and before enrolment in the study. The study was registered at the trial register of the Dutch Cochrane Centre under identifier 6090 and at the EU clinical Trials register with identification number 2015‐005258‐37 on 18 November 2015. The study was performed between March 2016 and March 2018. All procedures were performed in compliance with the current revision of the Declaration of Helsinki and Good Clinical Practice guidelines.

### 2.2 Patients

Forty patients with fibromyalgia (diagnosed by a rheumatologist) were recruited to participate in the study. Patients were suitable for inclusion if they had a pain score of at least 5 of 10 for most of the day and met the 2010 American College of Rheumatology diagnostic criteria (Wolfe et al., 2010). These criteria include a widespread pain index (WPI; 0–18 points), which defines the number of body areas in which a patient experienced pain during the last week and a symptom severity score (SyS‐score; 0–12 points), which indicates the presence and severity of other core symptoms of fibromyalgia such as fatigue, un‐refreshing sleep and cognitive symptoms. Patients were included if they either had a WPI ≥ 7 combined with a SyS‐score ≥ 5 or a WPI of 3–6 combined with a SyS‐score ≥ 9. Exclusion criteria included an age < 18 or > 75 years, a body mass index > 40 kg/m^2^, the presence of any medical disease (such as cardiovascular, pulmonary, renal, liver, cerebral or infectious disease), pregnancy or lactation, a history of psychosis, a history of illicit drug or alcohol abuse and the use of benzodiazepines. Patients were asked to stop any current pain medication for at least 4 weeks before the start of the study screening.

### 2.3 Study design

Patients visited the clinical research unit on five different occasions. The first visit was a screening visit where a physical examination was performed, baseline pain ratings were obtained and CPM and CCM tests were undertaken. In case of a normal physical examination and an absent or diminished CPM response (CPM response < 12%; see statistical analysis section for calculation), patients were included in the study. After inclusion, patients were randomized to either receive a 12‐week daily tapentadol sustained release (Grünenthal GmbH, Germany) or placebo treatment. Randomization was performed by a third party (independent investigator) using a computer‐generated randomization list. This list was transferred to the local pharmacy, which was responsible for dispensing of study medication. Tapentadol and placebo tablets were repackaged by the pharmacy for identical appearance. Treatment was started at a dose of 50 mg twice daily and weekly increased by 50–100 mg per day depending on the degree of pain relief and side effect profile to a maximum of 250 mg two times a day. In case of unacceptable side effects, dosages were decreased to a dose were side effects were acceptable. Visits 2, 3 and 4 were planned respectively 1, 2 and 3 months after the start of treatment. Visit 5 was planned 1 month after treatment ended. During these visits, CPM tests were performed and pain scores were obtained (using the 100‐mm visual analogue scale, VAS). Furthermore, patients were contacted on a weekly basis by telephone to query for pain scores and side effects.

### 2.4 Conditioned pain modulation

The CPM paradigm was performed as described previously (Niesters et al., 2014). In short, CPM was measured using heat pain as test stimulus and cold pain as conditioning stimulus (CS). Heat pain was applied on the volar side of the non‐dominant forearm with a 3 × 3 cm thermal probe connected to the Pathway Neurosensory Analyzer (Medoc Ltd., Ramat Yishai, Israel). During heat stimulation, patients rated the pain intensity level at the skin using a slider on a computerized potentiometer that ranged from 0 mm (no pain) to 100 mm (most intense pain imaginable), allowing for continuous monitoring of the visual analogue scale. At the start of each study day, the individual test temperature that induced a pain score between 50 and 60 mm was determined for each patient. For this, a series of heat stimuli were applied in which the temperature of the probe increased with 1.5°C/s from the baseline temperature (32°C) to a target temperature of various intensities (maximum 49°C) for 10 s after which the temperature returned to baseline (rate: 6°C/s). The target temperature that induced a VAS score between 50 and 60 mm was used during the remainder of the study day. Also for cold pain the individual test temperature was determined. Cold pain was induced using a cold water reservoir (Lauda, model Alpha RA8, Lauda‐Königshofen, Germany) that could be set to various temperatures (range 3–25°C). The foot and lower leg of the patient was immersed into the water reservoir and the patient rated the pain intensity of the cold water using the VAS. The temperature that induced a VAS score of 30–40 mm (on a scale from 0 to 100 mm) was used during the remainder of the study day.

CPM was measured at three different locations on the volar side of the non‐dominant forearm. On each location the VAS score of the test stimulus (heat pain) with and without the conditioning stimulus (cold pain) was determined using the slider of the computerized potentiometer. For the test stimulus, the temperature of the heat probe increased with 1.5°C/s from baseline (32°C) to the target temperature for 10 s after which the temperature rapidly returned (6°C/s) to baseline. The conditioning stimulus was applied 25 s before the start of the test stimulus and ended simultaneously with the test stimulus. Patients were specifically instructed to only rate the pain intensity level of the test stimulus.

### 2.5 Spontaneous pain ratings

To quantify spontaneous pain scores, the PainDetect questionnaire was used. This questionnaire is a screening tool to detect pain intensity and the presence of neuropathic pain symptoms. It assesses current pain intensity, pain intensity during the last 4 weeks, pain localization and pain qualification (i.e. burning, tingling, sharp and stubbing). Pain intensity was scored using the VAS, which comprises a 100 mm line where the left side end indicates no pain and the right end side indicates the worst pain imaginable. Patients were asked to mark the line at a point that corresponded with their pain intensity level. The PainDetect questionnaire includes a neuropathic symptom score that ranges from 0 to 38 points, were 0–12 points indicates the absence of neuropathic pain, 13–18 points indicates that a neuropathic component may be present and 19–38 points indicates that a neuropathic component is likely present.

### 2.6 Cornea confocal microscopy

CCM was performed on both eyes using the Rostock Cornea Module of the Heidelberg Retina Tomograph III (Heidelberg, Germany). After topical anaesthesia of both eyes the microscope was placed at the surface of the cornea apex and images were acquired with a 400 × 400 µm field of view and quantified using ACCmetrics software (provided by the faculty of Medical and Human Sciences of the University of Manchester, United Kingdom). Next, 3–10 representative, high‐quality images per eye were manually selected by a blinded investigator, which were used for quantification of the cornea nerve fibre length (CNFL), cornea nerve fibre density (CNFD) and cornea nerve branching density (CNBD). Each of these parameters for small nerve fibre pathology of the cornea were then compared to a reference value set published by Tavakoli et al. (2015). Small nerve fibre pathology was considered present if 2 of 3 parameters were defined as abnormal.

### 2.7 Sample size and statistical analyses

We did not perform a formal sample size analysis as no data were available on the efficacy of tapentadol on CPM in fibromyalgia patients. Based on the results of a previous study (Niesters et al., 2014), where patients were not selected on the absence of CPM prior to enrolment, the inclusion of 15 patients per group would result in a power > 90% to detect a 25% increase in CPM with a standard deviation of 20% for tapentadol treatment compared to placebo (alpha = 0.05, two‐tailed). We included an extra five patients per group to consider any margin of uncertainty around the effect size and *SD* and to compensate for expected drop outs due to the long study period.

All variables were screened for missing data, distribution abnormalities and outliers. Baseline characteristics were analysed with the appropriate parametric or non‐parametric tests. CPM responses were calculated using the area under the curve values of the electronic collected VAS data during the test stimulus with and without the conditioning stimulus. Averages of the three AUC responses per condition were calculated. To correct for variation in the magnitude of the responses between sessions and between subjects the relative CPM was calculated as: CPM% = [(mean AUC without CS − mean AUC with CS)/(mean AUC without CS)] × 100. The overall treatment effect (corrected for baseline) on the CPM% responses and the spontaneous pain scores (visits 1–4) were analysed using a mixed model with treatment as fixed effect and patient as random effect to account for repeated measurements over time. Similar analyses were performed on the CPM% responses and pain scores as function of CCM. The absolute pain scores were correlated to the CPM% responses and the neuropathic symptom score of the PainDetect questionnaire by Spearmans *ρ*. An analgesia responder rate analysis was performed to evaluate the proportion of patients who achieved predefined response rates in the range between 0% and 100%. The response rate was calculated by the proportion of pain relief compared to baseline measured at two time points during the treatment period (visits 2–4) compared to baseline. A Kolmogorov–Smirnov test was used to compare treatment distributions. CCM responses (normal vs. abnormal) between the different treatment responder groups and the correlation between CCM and the neuropathic symptom score of the PainDetect questionnaire were compared with a Fisher's exact test. A binary logistic regression was used to determine whether the CNFS was able to predict treatment responses where a treatment responder was defined by a reduction in pain reporting of at least 30%. SPSS (IBM Corp. Released 2017. IBM SPSS Statistics for Windows, Version 25.0. Armonk, NY: IBM Corp.) was used for all analyses, *p*‐values < 0.05 (two‐tailed) were considered significant. All data are reported as mean ± *SD* unless otherwise stated.

## 3 RESULTS

**Figure 1:**
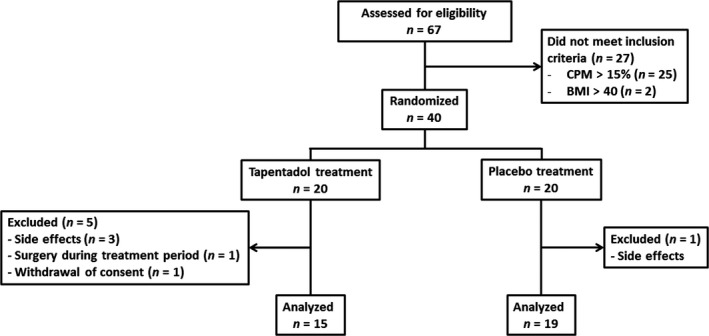
Flowchart of the study. BMI, body mass index; CPM, conditioned pain modulation

A total of 67 patients were assessed for eligibility of whom 27 were excluded because they did not meet the inclusion criteria; 40 patients were randomized to treatment. Six patients (five in the tapentadol group, one in the placebo group) did not complete the study period mostly due to unacceptable side effects. Since this occurred in the first weeks of treatment (before the measurement at month 1), analysis was performed only on the patients who completed the whole study period. See Figure 1 for the flowchart of the study. No significant differences were observed in baseline characteristics between the two study group. According to the PainDetect questionnaire, a neuropathic pain component was likely present in 60% of patients and possibly present in another 30% (Table 1). The average drug dose after the titration period was 340 ± 91 mg/day in the tapentadol group and 384 ± 129 mg/day in the placebo group. Side effects were reported in 14 of 15 patients in the tapentadol group and 14 of 19 patients in the placebo group. Reported side effects are listed in Table 2. Nausea was observed more frequently in patients treated with tapentadol (*p* = 0.005). Furthermore, although not significant, more patients in the tapentadol group reported opioid related side effects.

**Table 1:** Baseline characteristics

	Tapentadol group (*n* = 15)	Placebo group (*n* = 19)
Men/women (*n*)	1/14	1/18
Age (years)—median (range)	46.2 (23–62)	42.4 (24–67)
Weight (kg)—mean (*SD*)	78.7 (17.3)	81.8 (16.4)
Height (cm)—mean (*SD*)	1.70 (0.1)	1.72 (0.1)
Widespread Pain Index—mean (*SD*)	13.1 (3.0)	13.5 (3.1)
Symptom Severity Score—mean (*SD*)	9.2 (1.5)	8.8 (1.4)
Disease duration (years)	5.4 (4.9)	4.8 (3.8)
PainDetect		
Pain intensity score (mm)—mean (*SD*)	62.0 (13.1)	62.1 (14.0)
Neuropathic symptom score—mean (*SD*)	19.7 (6.5)	19.8 (5.8)
Score 13–18 (*n*, %)	5 (33.3)	5 (26.3)
Score 19–38 (*n*, %)	9 (60.0)	12 (63.2)

Abbreviation: *SD*, standard deviation.

**Table 2:** Number of patients reporting side effects

Side effect (*n*(%))	Tapentadol group (*n* = 15)	Placebo group (*n* = 19)	*p‐*value
Nausea	11 (73)	4 (21)	0.005
Dizziness	4 (27)	4 (21)	1.000
Headache	3 (20)	10 (53)	0.079
Dry mouth	1 (7)	0 (0)	0.441
Somnolence	4 (27)	1 (5)	0.146
Itch	4 (27)	1 (5)	0.146
Constipation	5 (33)	2 (11)	0.199
Shortness of breath	2 (13)	0 (0)	0.187
Palpitations	1 (7)	0 (0)	0.441
Weariness	3 (20)	2 (11)	0.634
Sweating	2 (13)	0 (0)	0.187
Depressive symptoms	2 (13)	0 (0)	0.187
Euphoria	1 (7)	0 (0)	0.441
Blurred vision	1 (7)	0 (0)	0.441
Muscle cramps	1 (7)	0 (0)	0.441

### 3.1 Conditioned pain modulation

**Figure 2:**
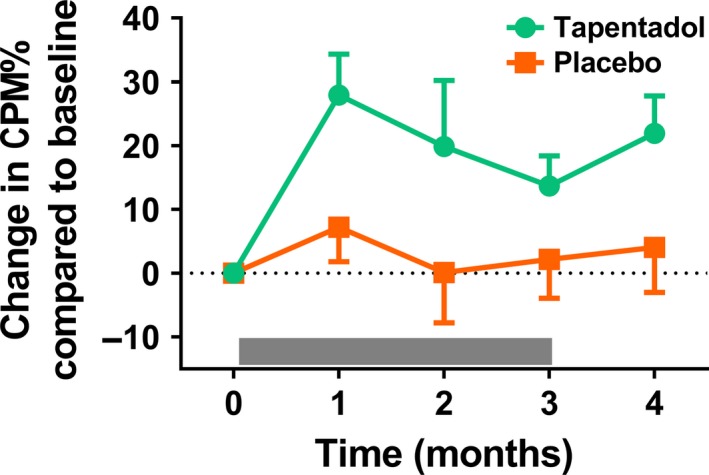
Change in CPM response relative to baseline before, during and after treatment with tapentadol (green circles) or placebo (orange squares). A significant treatment effect on CPM was observed for tapentadol compared to placebo (*p* = 0.042). The grey bar indicates the treatment period. Data are mean ± *SEM*. CPM, conditioned pain modulation

Average heat pain temperatures used to induce the CPM paradigm were 43.8 ± 3.1°C for the tapentadol group and 43.5 ± 2.4°C for the placebo group (*p* = 0.708), which induced pain scores of respectively 61.3 ± 17.4 mm and 57.9 ± 15.4 mm (*p* = 0.655). Average cold pain temperatures were for the tapentadol group 11.5 ± 6.3°C and 11.2 ± 6.7°C for the placebo group (*p* = 0.896). Corresponding pain scores were 40.0 ± 1.7 mm and 42.0 ± 1.9 mm respectively (*p* = 0.723). Before treatment no difference was observed in the magnitude of the CPM responses between the two treatment groups with a CPM% score of −4.0 ± 17.4% for the tapentadol group and 1.8 ± 13.9% for the placebo group (*p* = 0.297). CPM% responses increased during treatment with tapentadol with an average increase compared to baseline of 20.5 ± 12.5% for tapentadol compared to 3.0 ± 11.2% for placebo (mixed model visit 1–4: *p* = 0.042; see Figure 2).

### 3.2 Spontaneous pain scores

**Figure 3:**
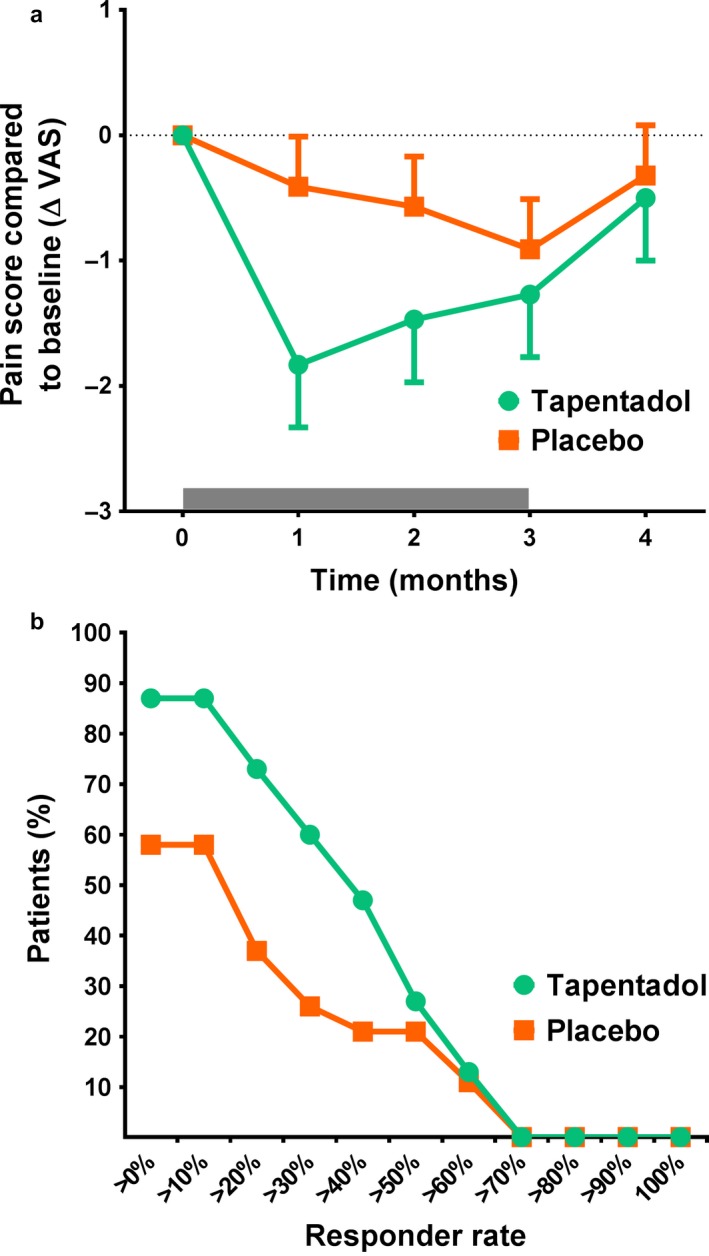
(a) Change in pain score compared to baseline before, during and after treatment with tapentadol (green circles) or placebo (orange squares). The grey bar indicates the treatment period. (b) Graph of the analgesia responder rate for predefined response rates in the range between 0% to 100%

Average spontaneous pain scores did not differ between the two groups before the start of treatment with a reported VAS score of 62.0 ± 13.1 mm for the tapentadol group and 62.1 ± 14.0 mm for the placebo group (*p* = 0.929). Overall, no significant difference in pain scores were observed during the 3‐month treatment period between tapentadol and placebo (mixed model visit 1–4: *p* = 0.115, Figure 3a). Analgesia responder rates for both treatments are plotted in Figure 3b for deciles ranging from 0% pain relief to 100% pain relief. This analysis demonstrated a treatment effect in favour of tapentadol (*p* = 0.007). No correlation was observed between pain scores and the neuropathic symptom score.

### 3.3 Analgesia versus CPM

**Figure 4:**
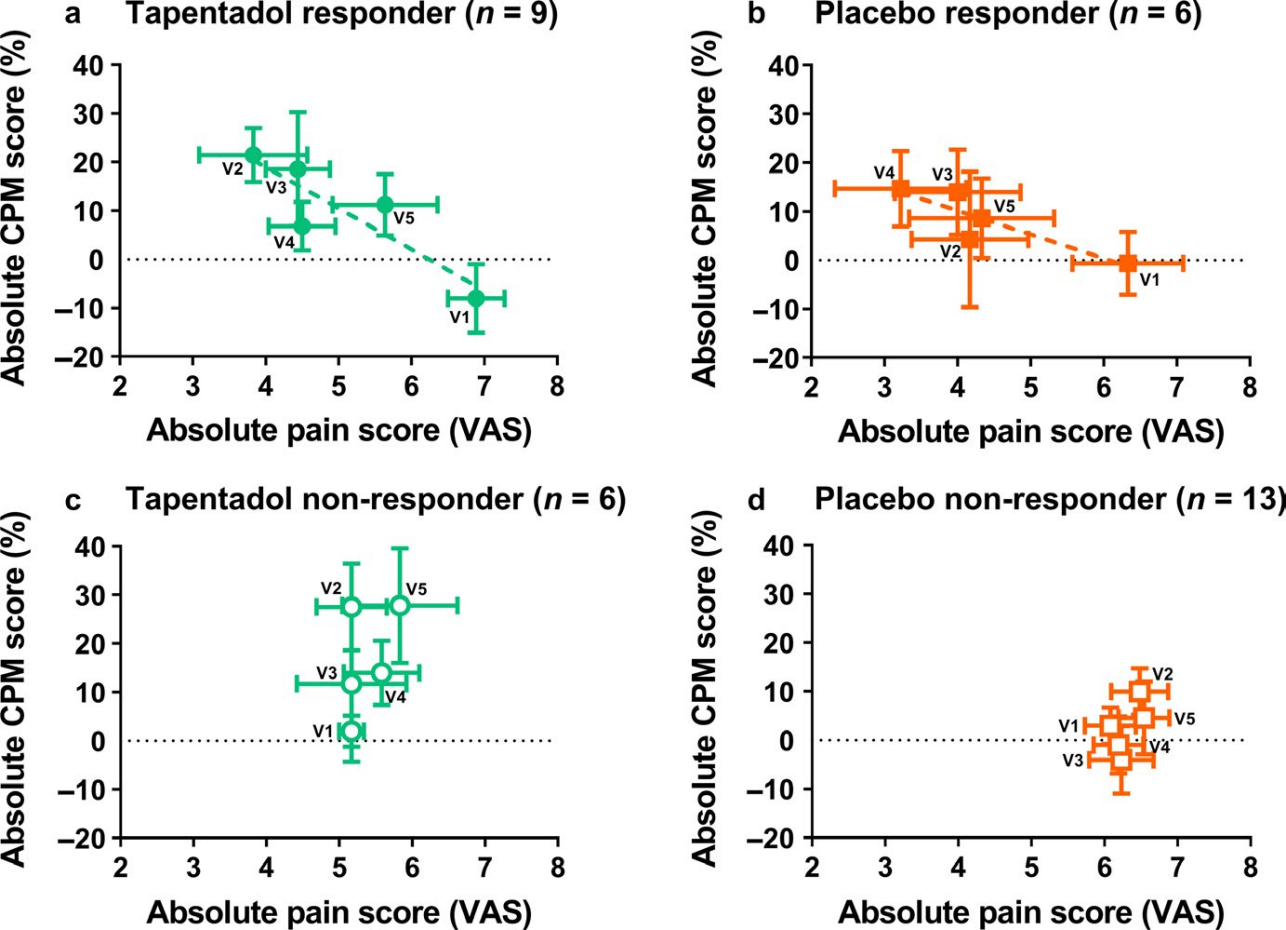
CPM% versus spontaneous pain ratings for the different visits in the (a) tapentadol responder group (closed green circles), the (b) placebo responder group (closed orange squares), the (c) tapentadol non‐responder group (open green circles) and the (d) placebo non‐responder group (open orange squares). A significant correlation was only observed for the tapentadol responder group (*r* ^2^ = 0.78; *p* = 0.047; green dotted line). Data are mean ± *SEM*. CPM, conditioned pain modulation; VAS, visual analogue scale; V1, visit 1; V2, visit 2; V3, visit 3; V4, visit 4; V5, visit 5

Patients with at least a 30% reduction of pain reporting were defined as either treatment or placebo responder. A highly significant correlation was observed between absolute pain scores and corresponding CPM% values per visit when all treatment responders (tapentadol and placebo) were taken together (*r* ^2^ = 0.60, *p* = 0.008). The correlation still existed when the treatment responder groups were analysed separately, but at a borderline significant level probably due to the smaller sample size (tapentadol responder group: *r* ^2^ = 0.78, *p* = 0.047; placebo responder group *r* ^2^ = 0.76, *p* = 0.055; Figure 4a,b). No correlation between absolute pain scores and CPM% values was observed in the non‐responder groups (Figure 4c,d). Overall, these data indicate that although tapentadol was able to increase CPM responses in a majority of patients this enhancement of CPM led to analgesia in only a subgroup of patients.

### 3.4 Cornea confocal microscopy

**Figure 5:**
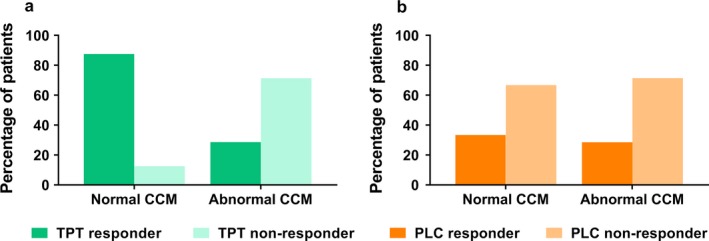
Percentage of patients with normal and abnormal cornea confocal microscopy images for the different responder groups in patients treated with (a) tapentadol or (b) placebo. CCM, cornea confocal microscopy; PLC, placebo; TPT, tapentadol

**Figure 6:**
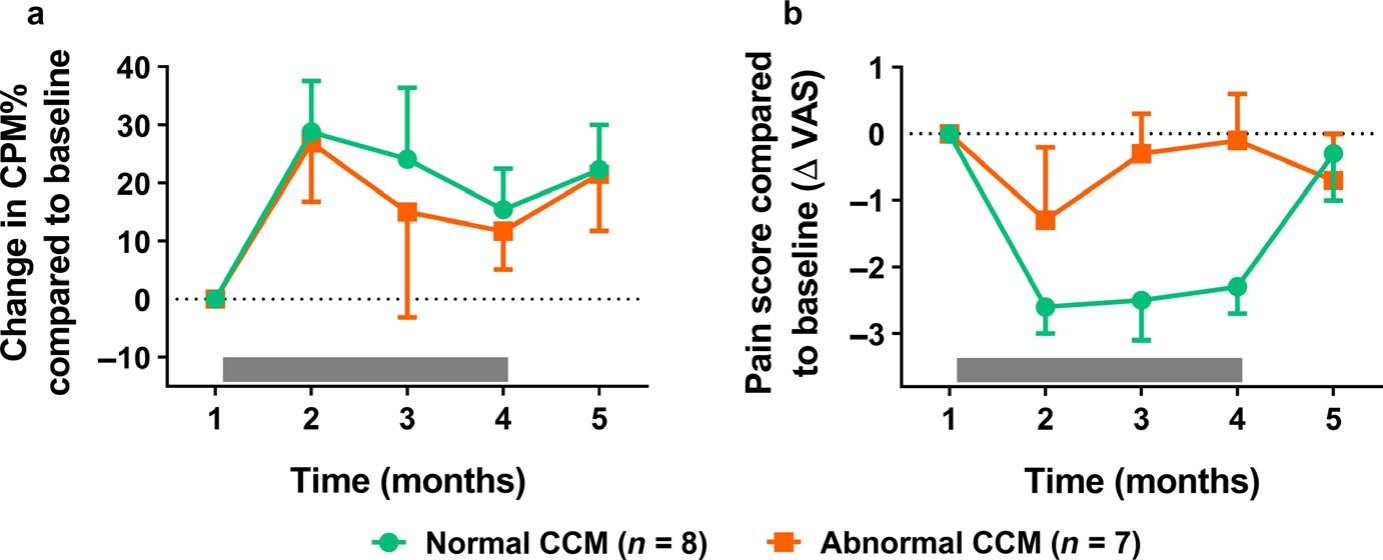
Change in CPM% (a) and pain scores (b) before, during and after treatment as function of cornea confocal microscopy images for the patients treated with tapentadol. The CPM response was not associated with the CCM status. Patients with normal CCM images displayed more pain relief, albeit not significant (*p* = 0.104). CCM, cornea confocal microscopy; CPM, conditioned pain modulation; VAS, visual analogue scale

Before the start of treatment each patient was photographed using CCM to evaluate the quantity and quality of the small nerve fibres in the cornea. Abnormal CCM images were observed in 38% of patients (see also Table 3). Interestingly, normal CCM images were observed in 87.5% of patients in the tapentadol responder group (eight of nine patients) compared to 12.5% of the patients in the tapentadol non‐responder group (one of six; *p* = 0.041). In the placebo group CCM images were similarly affected in both responder groups: 33.3% of the treatment responders (two of six) and 28.5% of the treatment non‐responders (4 of 13) had normal CCM images (Figure 5). Moreover, CCM was able to predict the treatment response in patients in the tapentadol group (*p* = 0.035) with a normal CNFS as predictor for tapentadol induced analgesia. CCM was not able to predict treatment effects in patients treated with placebo (*p* = 0.465). In Figure 6 the changes in CPM% and pain scores are presented as a function of CCM (normal vs. abnormal) for the patients treated with tapentadol. While the CPM response was not associated with the CCM status (Figure 6a), patients with normal CCM images displayed more pain relief, albeit not significant (Figure 6b; *p* = 0.104). No correlation was observed between CCM status and the neuropathic symptom score at baseline (*p* = 0.580).

**Table 3:** Cornea confocal microscopy

	All patients (*n* = 34)	TPT group (*n* = 15)	PLC group (*n* = 19)
Abnormal CCM (*n*, %)^a^	13 (38)	7 (47)	6 (32)
CNFD (*n*/mm^2^)	15.3	14.7	15.8
CNBD (*n*/mm^2^)	20.7	18.8	22.3
CNFL (*n*/mm^2^)	13.6	13.4	13.8

Abbreviations: CCM, cornea confocal microscopy; CNBD, cornea nerve branching density; CNFD, cornea nerve fibre density; CNFL, cornea nerve fibre length; PLC, placebo; TPT, tapentadol.

a Relative to reference value (Tavakoli et al., 2015).

## 4 DISCUSSION

In the current study, we evaluated the effect of a 3‐month tapentadol treatment on the endogenous pain modulatory system in fibromyalgia patients. In summary, we observed that tapentadol in contrast to placebo significantly increased the efficacy of the descending inhibitory pain pathway as measured by CPM. Patients with a normal cornea fibre state had an increase in CPM and displayed pain relief during tapentadol treatment. In contrast, patients with an abnormal cornea nerve state had no pain relief from tapentadol despite an increase in CPM.

The only earlier evidence in humans that tapentadol is able to increase descending pain inhibition comes from one previous study (Niesters et al., 2014). In this study, a 1‐month treatment period with tapentadol significantly increased CPM responses in patients with diabetic polyneuropathy. In agreement with that study, tapentadol also enhanced descending inhibition in the current study, which suggests a general beneficiary role of tapentadol in increasing CPM in multiple chronic pain conditions with absent or reduced CPM. Tapentadol is thought to increase CPM by its synergistic mode of action, which includes activation of the µ‐opioid receptor and noradrenaline reuptake inhibition (Zajączkowska et al., 2018). Both neurotransmitter systems are important in the activation of descending inhibition at supraspinal sites as well as at the level of the spinal cord dorsal horn (Ossipov et al., 2014). For example, microinjection of opioids in the periaqueductal grey, an important regulator of descending inhibition, produces powerful antinociception in animals. Furthermore, numerous animal studies showed that chemical and electrical stimulation of noradrenergic nuclei in the brain enhanced pain inhibition by release of norepinephrine into the cerebrospinal fluid. Spinally administered α_2_‐adrenergic agonists have been shown to induce antinociception in both animals and humans with a strong antinociceptive synergy when combined with opioids. See for an excellent review on this topic the study by Ossipov et al. (2014).

We studied fibromyalgia patients with absent or reduced CPM responses. About one‐third of fibromyalgia patients in our initial sample had relatively normal CPM responses (Figure 1). This, together with our observation of cornea nerve fibre abnormalities in a subset of patients, are indications of the large heterogeneity in the fibromyalgia patient population with respect to underlying pathophysiological mechanisms that determine development and/or maintenance of fibromyalgia symptoms, and additionally may explain the variations in success rate of pharmacological therapy. We a priori argued that patients with an absent or reduced CPM response were the most likely to benefit from treatment with tapentadol. Hence, a patient with a normal CPM response prior to treatment will likely still have a normal CPM response after treatment (i.e. a CPM response > 12%). Given our results, an important issue is whether the increase in CPM is the primary cause of the analgesic response or whether secondary conditions play an equally important role. The observation that not all subjects showed an analgesic effect from tapentadol despite a significant increase in CPM (Figure 5) points towards the later suggestion. For example, one factor for the successful translation of reactivated CPM into pain relief may be the CNFS (see below). Evidently, other yet unspecified factors may be involved as well.

The large heterogeneity within the fibromyalgia population, but also within other chronic pain syndromes, mandates a mechanism‐based approach when introducing pharmacological (and possibly also non‐pharmacological) treatment. As discussed earlier, it is important to stratify patients in homogeneous subgroups according to patterns of disease symptoms, (psycho)physical tests and underlying pathology (Cruz‐Almeida & Fillingim, 2014; Forstenpointer, Rehm, Gierthmühlen, & Baron, 2018; Oudejans et al., 2016; Oudejans, Niesters, Dahan, Brines, & Velzen, 2017). Specific symptoms or signs may then be used to predict treatment efficacy. Currently, there is some evidence for such predictive factors. For example, in post‐herpetic neuralgia patients, mechanical allodynia was associated with lidocaine efficacy (Attal, Rouaud, Brasseur, Chauvin, & Bouhassira, 2004), while heat pain thresholds correlated with opioid effect. Patients with chronic pancreatitis and a higher sensitivity to electrical stimulation in the pancreatic area were more likely to experience analgesia from pregabalin (Olesen et al., 2013). Also CPM is a known predictor of treatment efficacy. A less efficient CPM was correlated to duloxetine efficacy in patients with painful diabetic neuropathy (Yarnitsky, Granot, Nahman‐Averbuch, Khamaisi, & Granovsky, 2012), while patients with knee osteoarthritis and a more efficient CPM were more likely to show pain reduction during treatment with diclofenac (Edwards et al., 2016).

In the current study, we used CCM to phenotype patients according to their CNFS and showed that the nerve fibre condition was able to predict the tapentadol treatment effect. CCM is a relatively new technique to quantify small nerve fibres in the cornea. The technique has been validated in several patient populations with peripheral neuropathy in which good correlations were observed between the nerve fibre density in the cornea and in skin biopsies (Jiang et al., 2016; Petropoulos et al., 2013; Tavakoli et al., 2010). In the current study, tapentadol's analgesic efficacy was highest in patients with a normal CNFS. In fact, the responder rate was over 85% in this subset of patients (Figure 5). The reason for observed causality remains unknown. The abnormal CCM findings may be an indication of a more advanced disease state with a lesser sensitivity to treatment. Alternatively, we may have been studying two distinct patient phenotypes with a different sensitivity to treatment. An important observation is that, in line with previous findings, there was no correlation between CCM abnormalities and neuropathic symptom score of the PainDetect questionnaire (Oudejans et al., 2016). We do not know why these two indicators of neuropathy do not align in our fibromyalgia patient sample. Possibly, the development of pain symptoms and small fibre abnormalities are incongruent over the life cycle.

A limitation of the current study is the relatively small sample size. Still, we believe that due to the strict patient inclusion criteria our primary outcome measurements (enhancement of CPM) may be interpreted with reasonable reliability. The study was not powered to detect a difference in pain reporting and significant analgesic responses are not expected with these low numbers. Our secondary outcome measurement, the ability of the CNFS to predict treatment efficacy should be seen as hypothesis generating and needs further research in a large patient population to confirm or disclaim our findings. Another limitation of our study is that our conclusions are restricted to fibromyalgia patients with an impaired CPM response. It is of interest to assess tapentadol's analgesic efficacy in patients with fibromyalgia and normal CPM responses, and determine the role of the cornea nerve state on response efficacy.

Finally, it is important to discuss the use of tapentadol in this patient population in light of the current opioid epidemic. Tapentadol is a bifunctional analgesic at two distinct (one opioid and one non‐opioid) receptors to induce synergistic analgesic responses (Zajączkowska et al., 2018). It has a better safety index than classical opioid agonists such as oxycodone (Van der Schrier et al., 2017). We therefore do see some indication for its use in a subset of chronic pain patients, especially when treatment is restricted to periods no longer than 3 months, to allow an initial effective suppression of pain symptoms and most importantly to allow time to determine next treatment steps. However, our study suggests that the patients’ eligibility to such treatment may require phenotyping. Further studies are required to expand the process of phenotyping and assess whether phenotyping will lead to a reduction in the opioid adverse effects including (and most importantly) addiction and respiratory depression.

In conclusion, in the current study we demonstrate that a 3‐month treatment period with tapentadol significantly increased descending pain inhibition in fibromyalgia patients with reduced descending pain inhibition prior to treatment. Furthermore, tapentadol‐induced significant analgesia which was correlated to the magnitude of CPM increase, but only in patients with a normal CNFS.

## CONFLICT OF INTEREST

TvdD and AD received speakers’ fee from Grünenthal BV (Netherlands). All authors report no conflict of interest regarding the topic of this study.

## AUTHOR CONTRIBUTIONS

TvdD and MvV participated in patient management and data collection. TvdD, AD and MN performed the analyses. All authors discussed the study results and commented on the manuscript.
